# An Enzymatic Glucose Sensor Composed of Carbon-Coated Nano Tin Sulfide

**DOI:** 10.3390/nano7020039

**Published:** 2017-02-15

**Authors:** Ren-Jei Chung, An-Ni Wang, Shiuan-Ying Peng

**Affiliations:** Department of Chemical Engineering and Biotechnology, National Taipei University of Technology (Taipei Tech), Taipei 10608, Taiwan; dai20020223@gmail.com (A.-N.W.); terrypntut@gmail.com (S.-Y.P.)

**Keywords:** hydrothermal, chemical vapor deposition, carbon coated SnS (C-SnS), enzymatic glucose sensor

## Abstract

In this study, a biosensor, based on a glucose oxidase (GO*_x_*) immobilized, carbon-coated tin sulfide (SnS) assembled on a glass carbon electrode (GCE) was developed, and its direct electrochemistry was investigated. The carbon coated SnS (C-SnS) nanoparticle was prepared through a simple two-step process, using hydrothermal and chemical vapor deposition methods. The large reactive surface area and unique electrical potential of C-SnS could offer a favorable microenvironment for facilitating electron transfer between enzymes and the electrode surface. The structure and sensor ability of the proposed GO*_x_*/C-SnS electrode were characterized using scanning electron microscopy (SEM), X-ray diffraction (XRD), Raman spectroscopy, UV–vis spectroscopy, Fourier transform infrared spectroscopy (FTIR), and cyclic voltammetry study (CV).

## 1. Introduction

According to a global report on diabetes, published by the World Health Organization (WHO) in 2016 [1], more than 422 million adults (approximately) are living with diabetes, and its complications caused around 1.5 million deaths worldwide in 2012. Diabetes is a chronic disease; its syndrome is acknowledged by a high blood glucose (> 7.8 mM), due to insufficient control of hormones and endocrines in the human body. In order to prevent, or slow down the complications of diabetes, blood glucose must be regularly and accurately monitored. Biochemical tests are one of the most commonly-used techniques in detecting blood glucose, utilizing the oxidation of glucose and reduction of oxygen in blood in order to produce a linear current correlation with respect to glucose concentration [2,3,4]. Glucose biosensors, based on electrochemistry, can be divided into non-enzymatic and enzymatic types [5]. While the non-enzymatic type has great advantages, such as high sensitivity, high stability and the ability to work in extreme environments, such as in high temperatures and pH values, its specificity is limited and can be easily influenced by other interferents [5]. On the other hand, enzymatic sensors have high specificity and can be designed to fit individual chemicals, such as glucose oxidase (GO*_x_*) [3,4,6,7,8,9]. 

The selection of electrode materials is another key factor in order to improve performance by providing a large reaction area and a favorable microenvironment to facilitate electron transfer between enzymes and the electrode surface. More recently, the phase stability, band-structure, and optical properties of SnS have been studied intensively [10,11,12,13,14], and SnS holds an adjustable hole concentration, depending on the growth temperature and smaller band gap (~1.43 eV) compared with SnS_2_ (2.18–2.44 eV) [15]. Hence, SnS is suitable for use as a biosensor material. On the other hand, amorphous carbon has several interesting properties, such as good thermal conductivity, good mechanical strength, attrition resistance, is chemically inert, and possesses a satisfactory biocompatibility. The atomic structure is composed of a combination of sp^2^ and sp^3^ for carbon, which contributes to the electrical, optical, and mechanical properties [16,17]. Thereby, we propose a new enzymatic glucose biosensor, using carbon-coated SnS and GO*_x_*. In this study, we investigated the enzymatic glucose sensor, in which there is direct electron exchange between the enzyme and the electrode in order to complete the catalytic cycle. We prepared carbon-coated SnS (C-SnS) nanoflake powder using a simple two-step process, consisting of hydrothermal and chemical vapor deposition (CVD) methods. The structure and composition were identified using X-ray diffraction (XRD) and Raman spectroscopy, and the performance of glucose oxidase (GO*_x_*) on the C-SnS sensor was investigated by cyclic voltammetry study. 

## 2. Experimental Procedures 

The carbon-coated SnS (C-SnS) powder was synthesized using a two-step process. First, the SnS_2_ nanoflake was fabricated via the hydrothermal synthesis method in order to serve as the base material. An amount of 0.351 g of SnCl_4_, and 0.3 g of C_2_H_5_NS, were added into 70 mL of deionized water with 0.5 M NaOH_(aq)_ to adjust the pH value to 10.5; after stirring for 30 min, the solution was then heated to 200 °C and maintained for 12 h. In the following step, the precipitated SnS_2_ powder was then centrifuged, thoroughly washed, and dried at 80 °C for 12 h. A chemical vapor deposition (CVD) system was utilized for carbon coating, where ethanol was the source of carbon and was carried by Ar gas with a 50 sccm flow rate at 400 °C for 1 h. After carbon coating, the C-SnS was formed with a thick carbon layer; the possible formation mechanism for this will be discussed in this work. The structure of the as-synthesized SnS_2_ and C-SnS powders were characterized by X-ray diffraction and the θ/2θ scan ranged from 10° to 60°, where the SnS_2_ and SnS were identified according to joint committee on powder diffraction standards (JCPDS) cards No. 83-1705 and 75-0925, respectively. The microstructure of the SnS_2_ and C-SnS powders were observed from SEM topographies and selected area electron diffraction (SAED) patterns. Raman spectroscopy was used in order to identify the carbon coating of the C-SnS. 

The material stacking and assembling details of the GO*_x_*/C-SnS/GC electrode are as follows: 1 mg C-SnS powder and 1 mg GO*_x_* were mixed, separately, with 1 mL of deionized water, and then 5 μL of the individual solutions were transferred onto a polished, glassy carbon (GC) electrode on the order of C-SnS, and then GO*_x_*, and dried for 10 min at each step. Finally, 5 μL of 5% Nafion (Sigma-Aldrich) solution was used to coat and protect the GO*_x_* layer. The as-prepared electrode was immediately stored in a moisture resistant cabinet at room temperature, and the humidity was controlled below 50%. Prior to the glucose detection test, the adhesion of GO*_x_* onto C-SnS was examined using FTIR (HORIBA, Kyoto, Japan) and UV-vis spectroscopies (Agilent Technologies, Santa Clara, CA, USA). Three different working electrodes were used in order to compare with the GO*_x_*/SnS/GCE sensor; bare glassy carbon (GCE), C-SnS coating on GCE (C-SnS/GCE), and GO*_x_* on GCE (GO*_x_*/GCE) electrodes. As for the reference and auxiliary electrodes, the commercial Hg/Hg_2_Cl_2_ reference electrode and the platinum electrode were utilized, respectively. A potentiostat (CHI611E) was used for CV scanning and the potential scan ranged from –0.2 V to –0.7 V. First, the CV profiles of the different working electrodes were assessed in 0.1 M phosphate-buffered saline (pH = 6.0) in order to demonstrate the analytical performance of the GO*_x_*/C-SnS/GCE sensor. In order to further investigate the sensitivity and limits of the GO*_x_*/C-SnS/GCE glucose detector, different scanning speeds, from 50 mV/s to 300 mV/s, pH values, from 4 to 7, and glucose concentrations, from 0 to 1 mM, were used. Furthermore, the current vs. time curve of the GO*_x_*/C-SnS/GCE electrode was measured at –0.41V in 0.1 M phosphate-buffered saline (pH = 6.0) with respect to different glucose concentrations, from which the graph of corresponding current vs. glucose concentration was plotted and calibrated. Finally, the amperometric response of the GO*_x_*/C-SnS/GCE electrode to 0.1 mM glucose, as well as 0.1 mM interferents of citric acid (CA) and uric acid (UA), were measured in order to investigate the specificity in glucose censoring. In order to ensure the reproducibility, the fabrication has been carried out by different researchers in our group with triplicates, and several different biosensors, based on the proposed C-SnS, are still being investigated. 

## 3. Results and Discussion 

**Figure 1:**
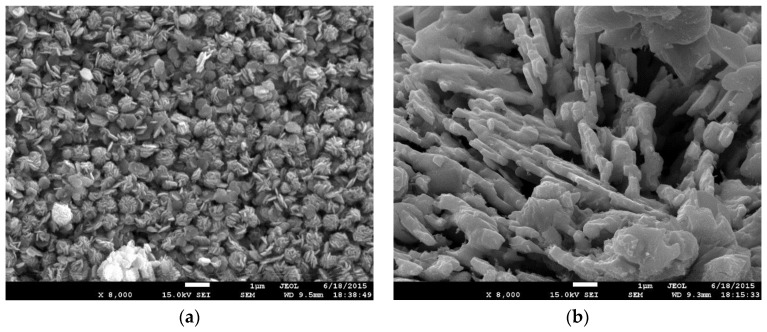
Scanning electron microscopy (SEM) images of (**a**) SnS_2_ and (**b**) carbon-coated tin sulfide (SnS) powders.

**Figure 2:**
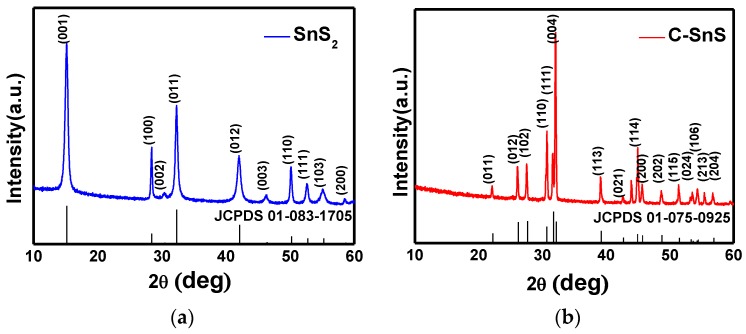
X-ray diffraction (XRD) θ/2θ scan of (**a**) SnS_2_ and (**b**) carbon-coated SnS powders.

**Figure 3:**
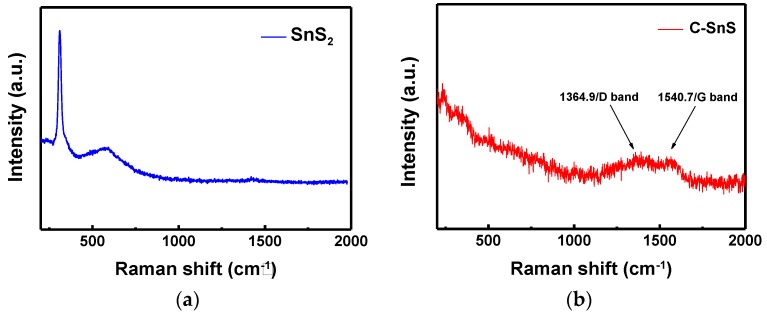
Raman spectrums of (**a**) SnS_2_ and (**b**) carbon-coated SnS powders.

The structures of the as-synthesized SnS_2_ and carbon-coated powders were observed from SEM images and XRD patterns, as depicted in Figure 1 and Figure 2, respectively. The SnS_2_ nanoflake powder size, observed from the SEM image, shows an average size of 500 nm in width with a thickness of 75 nm (Figure 1a); after carbon coating, the particle size increases dramatically and formed a laminar microstructure with a thickness of 200 nm (Figure 1b). From the XRD patterns in Figure 2a,b, corresponding to the as-synthesized SnS_2_ and the carbon-coated powders, both structures fit well with the JCPDS cards corresponding to hexagonal SnS_2_ and orthorhombic SnS crystalline structures; in addition, both possessed excellent crystallinity. The average domain sizes were 36 nm and 17 nm for SnS_2_ and SnS, respectively, calculated by the full-width-half-maximum (FWHM) at each diffraction peak using the Scherrer equation [18], and the results indicate an improvement in crystallinity from SnS_2_ to SnS. A small diamond carbon peak around 44° was found in Figure 2b and the existence of carbon can be further confirmed from the Raman spectrum (Figure 3), in which the D and G bands of carbon with I(D)/I(G)~1 appeared after carbon coating, while the Raman signal of SnS_2_ disappeared. The results of the Raman spectrum are consistent with those of the XRD patterns (Figure 2).

The transformation from SnS_2_ to SnS could be due to the phase stability of SnS*_x_* during the CVD process at 400 °C and also the reactive carbon gas environment [10,19]. According to the ab initio calculations by Burton et al. [19] and Vidal et al. [10], the SnS Pnma structure should be a preferable phase at 400 °C, which is consistent with our XRD and Raman observations. The excessive S during the phase transformation may assist the growth of amorphous hydrogenated carbon sulfur (a-C:H:S), and, thus, a clear D band was observed [16]. The enlarged laminar particle size and finer crystalline domain of SnS suggest that the SnS crystals were embedded in a preferentially-grown amorphous carbon, where the carbon layer grows faster along the surface (1–100) of the SnS_2_ nanoflake. The edge surface (1–100) of hexagonal SnS_2_ consisted of layered S and Sn atoms, which was able to promote the growth of a-C:H:S [16]. In sum, an obvious structural and compositional transformation, from hexagonal SnS_2_ to orthorhombic SnS, embedded in amorphous carbon with an improvement of crystallinity, could be achieved using a simple two-step synthesis process. 

**Figure 4:**
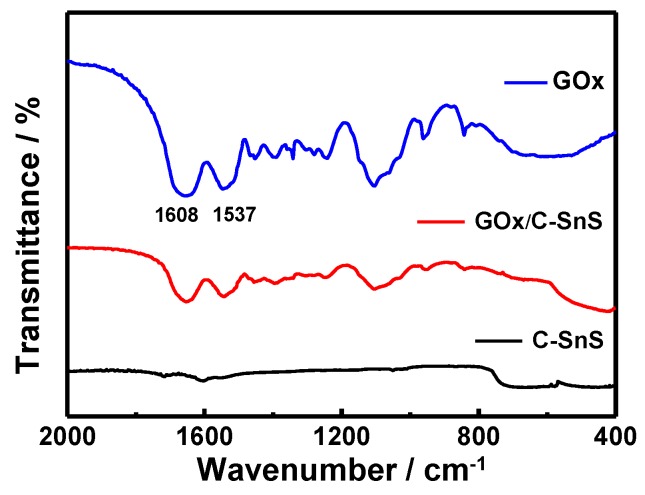
Fourier transform infrared spectroscopy (FTIR) of carbon-coated SnS (C-SnS), glucose oxidase (GO*_x_*), and GO*_x_* on C-SnS.

**Figure 5:**
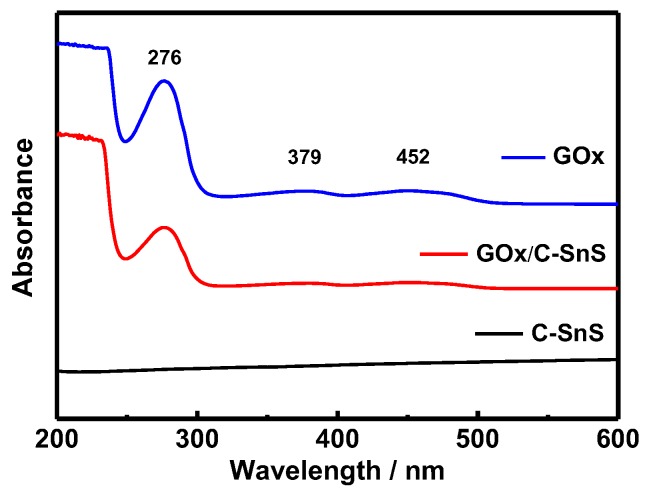
UV–vis spectroscopy of carbon-coated SnS (C-SnS), glucose oxidase (GO*_x_*), and GO*_x_* on C-SnS.

Prior to the glucose detection test, FTIR and UV-vis spectroscopies were used in order to examine the adhesion of GO*_x_* onto C-SnS, and the results are shown in Figure 4 and Figure 5. Without the GO*_x_* layer, the bare C-SnS did not react to FTIR and UV-vis due to the large wavelength compared to the lattice parameters of carbon and SnS. With respect to GO*_x_*, the Amide I and II bands can be observed from the FTIR pattern at 1537 cm^−1^ and 1608 cm^−1^ (Figure 4); additionally, the polypeptide chains at 276 nm and the oxidized form of the flavin groups in the proteins at 379 and 452 nm are also shown in the UV-vis pattern (Figure 5). From the FTIR and UV-vis patterns, the combination GO*_x_* on C-SnS shows the same characteristic signals as those mentioned above, indicating that the structure and activity of GO*_x_* remain the same in C-SnS.

**Figure 6:**
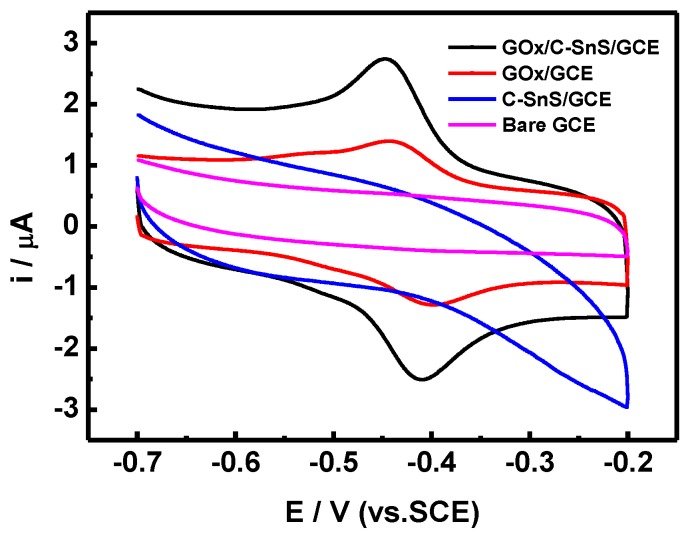
The cyclic voltammetry (CV) profiles of different working electrodes were used to compare with the GO*_x_*/SnS/GCE sensor, including bare glassy carbon electrode (GCE), C-SnS coating on GC (C-SnS/GCE), and GO*_x_* on GC (GO*_x_*/GCE) electrodes. The CV scan was carried out in 0.1 M PBS at a scan rate of 100 mV/s.

The results of the CV scans, using four different working electrodes, are shown in Figure 6. By adding C-SnS to GCE, the overall current range increases, but there is no obvious redox behavior. On the other hand, GO*_x_* shows a symmetric reduction and oxidation path, indicating that the redox behavior of GO*_x_* is a reversible reaction. With the assistance of C-SnS, the peak current of both the reduction and oxidation parts increased two times, and the applied potential was –0.41 V, which is comparable to results from the literature [20]. Hence, the GO*_x_*/C-SnS/GCE electrode shows a good redox behavior and, therefore, the stacking can serve as a glucose sensor.

**Figure 7:**
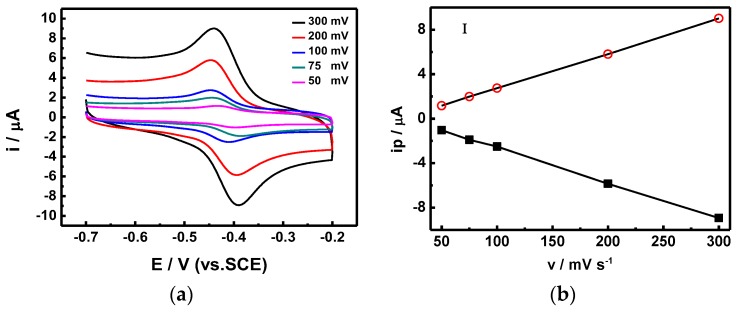
(**a**) CV profiles of the GO*_x_*/SnS/GCE sensor under different scan rates and (**b**) the resultant oxidation and reduction plots.

**Figure 8:**
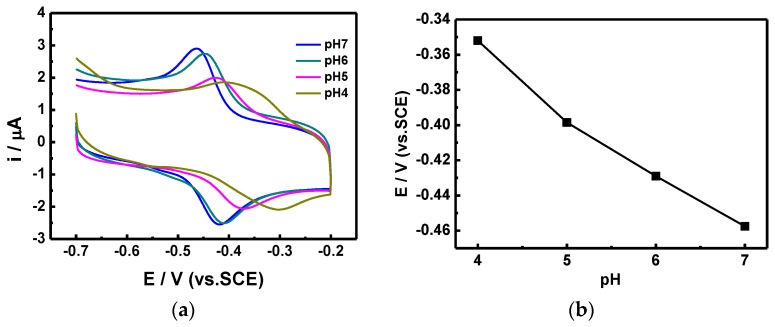
(**a**) CV profile of the GO*_x_*/SnS/GCE sensor under different pH values, and (**b**) the linear plotting of the E/V vs. pH values.

In order to calculate the electron transfer rate constant (Ks), the potential (Δ*E*_p_) difference between the reduction and oxidation was recorded using different scanning speeds (Figure 7), and calculated using Laviron’s equation [21]. The Ks of our proposed sensor stacking is 7.461 s^−1^, which is two times higher than those of similar glucose sensors (SnS_2_/GO*_x_*/GCE), proposed by Yang et al., which were measured at 3.68 s^−1^ [20]. Furthermore, the scanning rate showed a linear correlation with the redox current. We also utilized CV scans at different pH values (Figure 8) in order to optimize the sensing conditions. As the pH value decreased from 7 to 4, which is the working pH value of GO*_x_*, the reduction peak moved forward to a lower potential, and the corresponding current also decreased. The optimized response current occurred at pH = 7, which is the same pH value as that of human blood; hence, the proposed GO*_x_*/C-SnS/GCE sensor shows an excellent advantage in practical usage.

**Figure 9:**
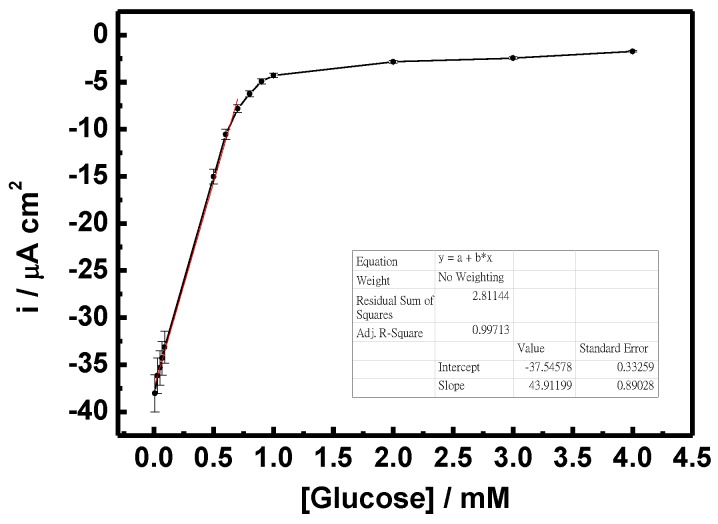
Current per unit area vs. glucose concentration of the GO*_x_*/SnS/GCE sensor.

**Figure 10:**
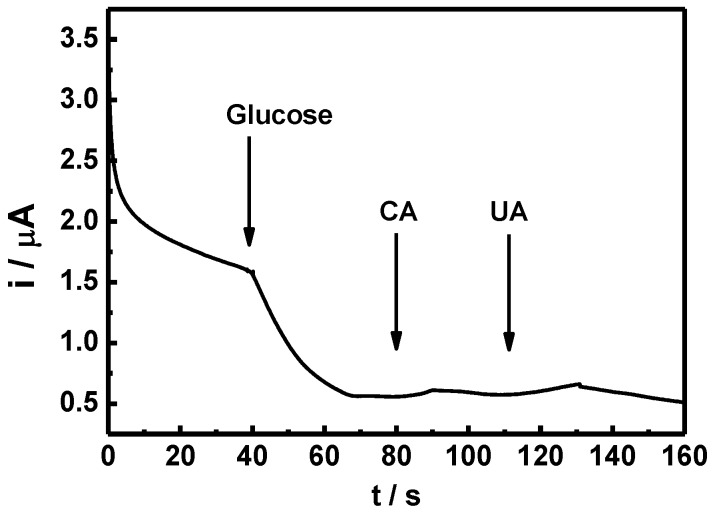
Amperometric response of the GO*_x_*/SnS/GCE electrode to 0.1 mM glucose, as well as 0.1 mM interferents of uric acid (UA) and citric acid (CA).

**Figure 11:**
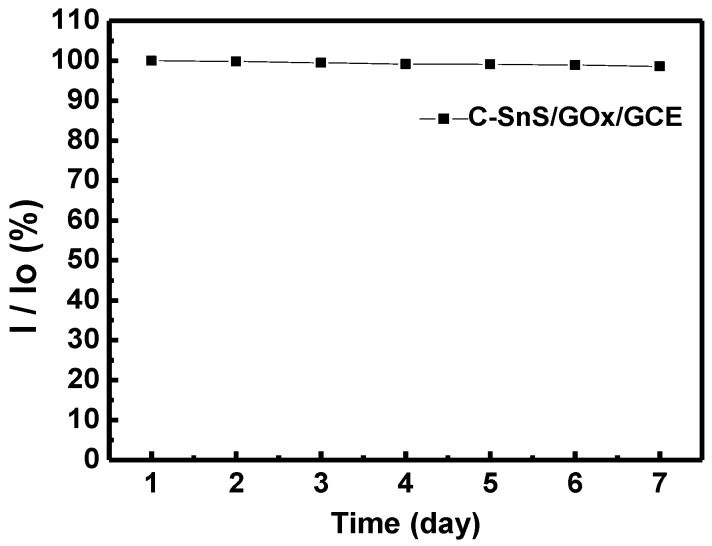
The performance stability of the C-SnS/GO*_x_*/GCE sensor for 7 days, where 0.1 mM glucose was used.

Figure 9 shows the sensitivity of the GO*_x_*/C-SnS/GCE sensor with respect to different glucose concentrations. According to the results of triplicates, the relative standard deviation (RSD) was less than 5.2% calculated from the current response of freshly prepared electrodes. The results convinced that the fabrication method was highly reproducible comparing with reported enzymatic and non-enzymatic glucose sensors [22,23]. A linear correlation, from 0.03 to 0.7 mM, can be found in glucose concentrations, and the sensitivity was 43.9 mA·M^−1^·cm^−2^, roughly six times that found in Yang’s work, using SnS_2_ [8]. Since human blood always contains different hormones and chemicals, which can interfere glucose sensor detection, an amperometric test was carried out using CA and UA, as depicted in Figure 10. Although the reactions are not clear, only a minor reaction occurred with the two interferents, which are in an acceptable and distinguishable response range. The amperometric response of the GO*_x_*/SnS/GCE electrode demonstrates an acceptable selectivity to glucose. For enzymatic glucose sensor, it is reported that the retention of the original response may drop to less than 76% within seven days [24]. Furthermore, long-term stability was studied, and the results are provided in Figure 11. A stable and reproducible current over seven days was observed in our investigation. The presently commercialized glucose sensors are designed for blood samples. The users have to use lancet or syringe to collect blood from finger pricking or phlebotomizing. Noninvasive routes for glucose monitoring are highly expected to prevent these disadvantages. Thus, succedaneous body fluid samples such as urine, tear or saliva become prevailing targets for novel glucose sensor design. Proportional to blood glucose, however, they have relatively low glucose content [25]. A more sensitive glucose sensor with lower detection limit is required, and the developed sensor in this study will benefit. As to human blood, nevertheless, lower detection range needs fewer samples with proper dilute design on the system. 

## 4. Conclusions 

In this work, we reported a C-SnS nanoparticle powder with a laminar structure for use in enzymatic glucose sensor applications via a simple two-step synthesis process, using hydrothermal and chemical vapor deposition methods. The preparation of the proposed sensor is easy and cost-efficient. The fast electron transformation rate (Ks = 7.46 s^−1^), high sensitivity (43.9 mA·M^−1^·cm^−2^), linear range from 0.03 to 0.7 mM glucose, and acceptable selectivity show promising development potentials for glucose sensing.
